# Supplementary material for: Identification of B-Cell Linear Epitopes in the Nucleocapsid (N) Protein B-Cell Linear Epitopes Conserved among the Main SARS-CoV-2 Variants
Source: Viruses. 2023 Apr 6;15(4):923. doi: 10.3390/v15040923 (PMC10145278; doi:10.3390/v15040923)
Supplement: Supplementary file 1 [file viruses-15-00923-s001.zip › viruses-2318237-supplementary.pdf]

**Table S1: Frequency of N protein key mutations across the SARS-CoV-2 major variants.**

| Variant | Mutations | | | | | | | | | | | | | | | |
|---|---|---|---|---|---|---|---|---|---|---|---|---|---|---|---|---|
| | D3L | Q9L | P13L | Del31/33 | D63G | P80R | E136D | R203M | R203K | G204R | T205I | G215C | L230F | S235F | D377Y | S413R |
| Alpha | 98,2% | 0,1% | 0,1% | | 0,1% | | 0,2% | 0,1% | 98,1% | 91,1% | 0,1% | 0,1% | 0,1% | 98,9% | 0,1% | |
| Beta | 0,1% | 0,1% | 0,6% | | 0,1% | 0,1% | 0,1% | 0,1% | 0,1% | 0,1% | 97,8% | 0,1% | 0,1% | 0,1% | 0,2% | |
| Gama | 0,1% | | 0,1% | | 0,1% | 99,2% | 0,1% | 0,1% | 95,2% | 95,6% | 0,2% | 0,1% | 0,1% | 0,1% | 0,7% | |
| Delta | 0,1% | 7,2% | 0,1% | | 97,3% | | 0,1% | 99,0% | 0,1% | 0,1% | 0,1% | 89,3% | 0,3% | | 98,1% | 0,1% |
| Omicron | 0,1% | | 97,9% | 91,9% | 0,1% | | 6,5% | 0,1% | 97,8% | 98,0% | 0,1% | 0,1% | 0,1% | | 0,1% | 64,2% |
| XBB.15* | | 0,1% | 99,6% | 97,9% | 0,1% | 0,1% | 0,1% | | 98,9% | 98,8% | 0,1% | | 0,1% | | 0,1% | 98,2% |
| CH.1.1 | | | 99,4% | 96,1% | | 0,1% | 0,1% | | 98,2% | 98,3% | 0,1% | | 0,1% | | | 98,2% |
| BA.2.75* | | | 99,3% | 92,7% | 0,1% | | 0,1% | | 98,5% | 98,5% | 0,1% | | 0,1% | | | 98,0% |
| BA.5* | | | 98,1% | 86,1% | 0,1% | | 22,2% | | 98,2% | 98,3% | 0,1% | | 0,1% | | | 98,2% |
| BQ.1* | | 0,1% | 99,5% | 96,4% | 0,1% | | 98,6% | | 98,7% | 98,7% | 0,1% | | 0,1% | | | 98,6% |
| BQ1.1. | | 0,1% | 99,5% | 96,2% | 0,1% | | 98,6% | | 98,7% | 98,8% | 0,1% | | 0,1% | | | 98,6% |
| XBB.1 | 0,1% | 0,1% | 98,7% | 94,4% | | | 0,1% | | 99,3% | 99,3% | 0,1% | | 0,1% | | | 98,1% |
| XBB.1.5 | | 0,1% | 99,6% | 97,9% | 0,1% | | 0,1% | | 99,1% | 99,0% | 0,1% | | 0,1% | | | 98,1% |
| CH.1.1.1 | | | 99,3% | 93,2% | | | 0,1% | | 98,8% | 98,9% | 0,1% | | 0,1% | | | 98,0% |
| BM.4.1 | | | 100,0% | 77,9% | | | | | 97,4% | 98,7% | | | | | | 94,8% |
| BM.4.1.1 | | | 98,7% | 87,7% | | | | | 99,0% | 99,0% | | | | | | 99,0% |
| XBB.1.9 | | | 98,9% | 94,8% | | | | | 98,3% | 98,4% | | | | | | 95,8% |
| B.1.617.2 | 0,1% | 0,1% | 0,1% | 0,1% | 93,2% | 0,1% | 0,1% | 97,2% | 0,1% | 0,1% | 0,1% | 67,4% | 0,2% | 0,1% | 0,1% | 0,1% |
| P.1.1 | 0,8% | | | | 0,1% | 99,2% | | 0,2% | 68,8% | 75,7% | 0,1% | | 0,2% | 0,1% | 1,0% | |
| BA.2 | 0,1% | | 98,8% | 95,2% | 0,1% | | 0,1% | 0,1% | 96,9% | 97,0% | 0,1% | 0,1% | 0,1% | | | 97,5% |
| AY.43 | 0,1% | 98,6% | 0,1% | 0,1% | 95,6% | | 0,1% | 99,3% | 0,1% | 0,1% | 0,1% | 99,2% | 0,1% | 0,1% | 98,8% | |
| AY.34.1 | | 0,1% | 0,1% | | 97,5% | | | 99,0% | 0,1% | 0,1% | | 99,1% | 98,4% | 0,1% | 96,9% | |

The table indicates the frequencies of 16 key mutations described in the SARS-CoV-2 N protein (columns) across the previously Circulating Variants of Concern (Alpha, Beta, Gamma, and Delta), the Current Variants of Concern (Omicron, Omicron - XBB.1.5.X, Omicron – CH.1.1.X, Omicron – BA.2.75.X, Omicron – BA.5, Omicron BQ.1.X, BQ1.1, and XBB.1), the top 5 growing lineages in the world on 2023 February 27$^{th}$ (XBB.1.5, CH.1.1.1, BM.4.1, BM.4.1.1, XBB.1.9) and the top five most counted lineages in Brazil (B.1.617.2, P.1.1, BA.2, AY.43, AY.34.1). Values represent the frequency of each mutation among all reported isolates.
